# Influence of glycemic control on gain in VO2 peak, in patients with type 2 diabetes enrolled in cardiac rehabilitation after an acute coronary syndrome. The prospective DARE study

**DOI:** 10.1186/s12872-015-0055-8

**Published:** 2015-07-08

**Authors:** Bruno Vergès, Bénédicte Patois-Vergès, Marie-Christine Iliou, Isabelle Simoneau-Robin, Jean-Henri Bertrand, Jean-Michel Feige, Hervé Douard, Bogdan Catargi, Michel Fischbach, DARE Study group

**Affiliations:** 1 Service endocrinologie, diabétologie, CHU Le Bocage, 21000 Dijon, France; 2 Unité de réadaptation cardiaque, Clinique Les Rosiers, Dijon, France; 3 Service réadaptation cardiaque, Hôpital Corentin Celton, Issy les Moulineaux, France; 4 Unité de réadaptation cardiaque, Clinique du Lavarin, Avignon, France; 5 Service réadaptation cardiaque, Hôpital du Haut Lévêque, Pessac, France; 6 Service endocrinologie, diabétologie, Hôpital du Haut Lévêque, Pessac, France; 7 URCA, Bordeaux, France

**Keywords:** Diabetes, Cardiac rehabilitation, Myocardial infarction, Hyperglycemia

## Abstract

**Background:**

Gain in VO2 peak after cardiac rehabilitation (CR) following an acute coronary syndrome (ACS), is associated with reduced mortality and morbidity. We have previously shown in CR, that gain in VO2 peak is reduced in Type 2 diabetic patients and that response to CR is impaired by hyperglycemia.

**Methods:**

We set up a prospective multicenter study (DARE) whose primary objective was to determine whether good glycemic control during CR may improve the gain in VO2 peak. Sixty four type 2 diabetic patients, referred to CR after a recent ACS, were randomized to insulin intensive therapy or a control group with continuation of the pre-CR antidiabetic treatment. The primary objective was to study the effect of glycemic control during CR on the improvement of peak VO2 by comparing first the 2 treatment groups (insulin intensive vs. control) and second, 2 pre-specified glycemic control groups according to the final fructosamine level (below and above the median).

**Results:**

At the end of the CR program, the gain in VO2 peak and the final fructosamine level (assessing glycemic level during CR) were not different between the 2 treatment groups. However, patients who had final fructosamine level below the median value, assessing good glycemic control during CR, showed significantly higher gain in VO2 peak (3.5 ± 2.4 vs. 1.7 ± 2.4 ml/kg/min,p = 0.014) and ventilatory threshold (2.7 ± 2.5 vs. 1.2 ± 1.9 ml/kg/min,p = 0.04) and a higher proportion of good CR-responders (relative gain in VO2 peak ≥ 16 %): 66 % vs. 36 %, *p* = 0.011. In multivariate analysis, gain in VO2 peak was associated with final fructosamine level (*p* = 0.010) but not with age, gender, duration of diabetes, type of ACS, insulin treatment or basal fructosamine.

**Conclusions:**

The DARE study shows that, in type 2 diabetes, good glycemic control during CR is an independent factor associated with gain in VO2 peak. This emphasizes the need for good glycemic control in CR for type 2 diabetic patients.

**Trial registration:**

Trial registered as NCT00354237 (19 July 2006).

## Background

Several studies have clearly shown that cardiac rehabilitation (CR) significantly reduces cardiovascular morbidity and mortality and improves quality of life. The clear benefit of CR on overall mortality and cardiovascular mortality has been confirmed by several clinical trials [1, 2], meta-analyses [3–8] and population-based surveillance studies [9]. The cardiovascular mortality rate in patients who underwent CR with exercise training after MI was found to be 20 % to 26 % lower than in those who did not attend a CR program [3, 4, 8]. Long-term reduction in cardiac and total mortality after CR was confirmed by *Hedbäck et al.*, who showed a 26.7 % reduction in total mortality and a 27.1 % reduction in cardiovascular mortality over a 10-year period [10]. Hence, CR programs are recognized as an integral part of the care strategy for patients with coronary heart disease, heart failure, cardiac surgery and peripheral artery disease, and CR is a class I recommendation in patients with coronary heart disease [11–15].

Exercise capacity is an independent factor reducing overall and cardiovascular mortality [16]. One of the results of CR is to improve exercise capacity (assessed on peak oxygen uptake [peak VO2]), in patients with established coronary heart disease. It has been reported, based on a long-term prognosis study in 12 169 men in CR, that exercise capacity, determined by direct measurement of peak VO2, exerts a major long-term influence on prognosis in patients after Myocardial Infarction (MI) or ischaemic heart disease, with a 9 % improvement in prognosis for 1-ml/kg/mn increment in peak VO2 [17]. *Vanhees et al.* have demonstrated that peak VO2 improvement after CR reduced significantly cardiovascular morbidity and mortality in coronary patients [18]. All these data indicate that a part of the benefit of CR on cardiovascular morbidity and mortality is related to VO2 improvement.

It is well established that cardiovascular disease is the major cause of morbidity and mortality in patients with type 2 diabetes and that coronary disease risk is 2–4 fold increased over non-diabetic subjects [19]. In addition, prognosis after MI is worse in diabetic patients than in non-diabetic subjects [20]. Due to their high cardiovascular risk, patients with type 2 diabetes are highly recommended for CR. However, several studies have shown that CR is less effective in patients with diabetes [21–24]. Our group has previously shown that gain in VO2 peak in CR, after an acute ischaemic heart event, was significantly less in patients with type 2 diabetes than in non-diabetic patients (13 % vs. 30 %, p = 0.002) [24]. In addition, we found an inverse relation between fasting blood glucose and change in VO2 peak on both univariate (r = −0.40, p = 0.002) and multivariate (p = 0.001) analyses suggesting that response to CR may be impaired by poor glycemic control [24].

This prompted us to set up a prospective multicenter study (the DARE [Diabetes in cArdiac REhabilitation] Study) in order to determine whether good glycemic control during CR may improve the gain in VO2 peak.

## Methods

### Patients

We recruited 64 type 2 diabetic patients enrolled in CR early after a recent (in the 4 previous weeks) acute coronary syndrome (ACS), treated by percutaneous coronary intervention. Type 2 diabetes was defined according to the American Diabetes Association criteria (at least 2 fasting blood glucose values above 7.0 mmol/L or 126 mg/dl). All patients had an HbA1c level above 7 % at time of referral to CR.

Patients who had been treated by coronary bypass surgery, with renal failure (glomerular filtration rate below 30 ml/min), severe peripheral arterial disease (Leriche-Fontaine stage ≥ 3) severe respiratory failure and those unable to perform exercise testing and training were not included in the study.

Before starting the CR program, the patients were randomized either in an intensive treatment group with basal-bolus insulin therapy or in a control treatment group in which the antidiabetic treatment at time of enrolment in the study was maintained.

In the intensive treatment group, patients received a daily injection of basal insulin glargine (Lantus®) administered before dinner and an injection of a rapid-acting insulin analogue aspart (Novorapid®) bolus before each meal. The insulin regimen was set up by a diabetologist before the beginning of the CR program. The initial basal insulin dose was calculated according to body weight, gender and fasting blood glucose using the algorithm recommended by *Holman* and *Turner* [25, 26]. The initial bolus prandial dose was 1/3 of the basal insulin dose. The patients of the intensive treatment group were asked to perform self monitoring of blood glucose (SMBG) 6 times a day (pre and post breakfast, pre and post lunch, pre and post dinner). During the study, titration was performed according to the algorithm from the “treat to target” trial [27] for the basal insulin dose and according to the post-prandial blood glucose values for the prandial bolus insulin doses. In this patient group the capillary blood glucose targets have been defined according to the IDF/Europe recommendations: fasting capillary glucose <1.00 g/l (5.5 mmol/l) and post-prandial capillary glucose <1.35 g/l (7.5 mmol/l) [28]. Education on diabetes, including insulin injection and SMBG, was performed in all the patients from the intensive treatment group. A diabetologist was contacted at least once a week for the patients of the intensive treatment group. Patients from the intensive treatment group were also allowed to be treated with metformin on top of insulin when all other antidiabetic treatments were stopped during the study.

In the control group, the antidiabetic treatment at time of enrolment in the study was maintained. During the CR period, modification of the antidiabetic treatment was permitted in order to avoid hypoglycemic events or major hyperglycemias.

The primary objective of the DARE study was to examine the effect of strict glycemic control during CR, following an ACS, on the improvement of peak VO2. For this purpose, we planned to compare prospectively the improvement of peak VO2 during CR first, between the patients randomized in the intensive insulin treatment group versus the patients randomized in the control treatment group, second between the patients who showed good glycemic control during the CR period (final fructosamine below the median value) versus those who showed unsatisfactory glycemic control during the CR period (final fructosamine above the median value). The secondary objectives were to evaluate the effect of strict glycemic control during cardiac rehabilitation on the improvement of ventilatory threshold and on the number of patients considered as “CR responders” (showing a relative gain in VO2 peak ≥ 16 %).

Each subject gave written informed consent before participation in the study, which has been approved by the Dijon Ethics Committee and has been registered (trial registered as NCT00354237).

### Cardiac rehabilitation program

At the beginning and at the end of the CR program, each patient underwent a cardiopulmonary exercise test performed on a cycle ergometer. This symptom limited cardiopulmonary exercise test started with an initial workload of 20 watts with increments of 10 watts at each 1-min exercise stage. During that test, peak VO2 (ml/kg/min) and ventilatory threshold (ml/kg/min) were measured. Ventilatory and gas exchange data were determined on a breath-by-breath basis with a computerized system. Peak oxygen consumption (peak VO2) was defined as the highest consecutive 30-s averaged value obtained during exercise test [29]. Ventilatory Threshold was estimated by the Wasserman method as the point where ventilatory equivalent ratio for oxygen (VE/VO2) starts to increase without concomitant increase in the ventilatory equivalent ratio for carbon dioxide (VE/VCO2) [30].

The CR program, typical of a post-ACS CR program, consisted in 20 physical training sessions realized in a period shorter than 8 weeks. Each session included a 30 min period on bicycle with a training heart rate (HR) corresponding to the HR obtained at the first ventilatory threshold during the initial bicycle exercise test according to the usual CR recommendations [12]. Moreover, during therapeutic training, exercise intensity target was also based on the rating of perceived exertion (RPE), usually between 12 and 14 on the Borg’s scale [14]. All the patients were informed how to use the Borg’s scale before training. Each session was supervised by a skilled team including cardiologists, cardiovascular nurse specialists and exercise physiologists. All the patients included in the DARE study had a similar CR program.

As usually accepted, patients were considered as “CR-responder” when their relative increase in peak V02 after CR was equal or more than 16 % [31].

### Metabolic evaluation

At the beginning and at the end of the CR program, fasting blood samples were collected for evaluation of fasting glucose, HbA1c and fructosamine. Fructosamine is an estimate of mean glycemic level during the 2–3 previous weeks [32], when HbA1c is an estimate of mean glycemic level during a much longer period of 3 months. Thus, because the CR program was limited to a 4–8 week period of time, fructosamine at the end of the CR program, instead of HbA1c, was used to assess mean glycemic level during CR.

### Statistical analysis

Data are expressed as means ± Standard Deviation. Statistical calculations were performed using the SPSS software package (SPSS Inc., Chicago IL, USA). Comparisons of means between groups were performed by two-tailed Student’s *t*-test. Comparisons of percentages between groups were performed by Chi-2 test. When comparing percentages with an expected count smaller than 5 in the 2 × 2 contingency table, the Chi-2 with the Yates correction was used. Baseline and post-cardiac rehabilitation data were compared by paired *t*-test. The correlation coefficients (r) were determined by linear regression analysis. Statistical significance of the correlation coefficients was determined by the method of Fisher and Yates. Multivariable analysis was performed by stepwise linear regression for continuous dependent variables or by stepwise logistic regression for dichotomous dependent variables. A p value <0.05 was considered statistically significant.

## Results

### Baseline characteristics

Among the 64 patients included in the study, 57 completed the study. The drop out of 7 patients was due to personal reasons and occurred during the initial period of the CR program. The baseline data of the patients who completed the study, in each treatment group (intensive treatment group, control treatment group) are shown in Table 1. No significant differences were seen between the 2 groups for gender, age, diabetes duration, history of previous Coronary Heart Disease (CHD), tobacco smoking, BMI, type of ACS, resting heart rate, systolic and diastolic blood pressure, use of antidiabetic agents at time of inclusion in the study (metformin, sulfonylurea or glinide, acarbose, pioglitazone, DPP-4 inhibitor, insulin), use of cardiovascular drugs (ACE inhibitor/ARB, betablocker, antiplatelet agent, statin), baseline values of HbA1c, fructosamine, ventilatory threshold and VO2 peak. As far as baseline antidiabetic treatment is concerned, no differences between intensive treatment group and control treatment group were observed for insulin program: one daily basal insulin injection (6 vs. 6), two daily pre-mixed insulin injections (4 vs. 6) and more than 3 daily injections (5 vs. 5). No patients were on insulin pump. As far as oral antidiabetic drugs (OAD) are concerned, the number of patients on one OAD, 2 OADs and 3 or more OADs were 10, 7, 0 and 8, 8, 2 in the control group and the insulin intensive treatment group respectively (NS).

**Table 1:** Baseline characteristics

	Control treatment group (n = 31)	Intensive treatment group (n = 26)	
Gender (M/F)	24/7	22/4	NS
Age (yrs)	58 ± 10	60 ± 10	NS
Diabetes duration (yrs)	7 ± 6	9 ± 8	NS
History of previous CHD	9 (29 %)	6 (23 %)	NS
Tobacco smoking n(%)	10 (32 %)	8 (31 %)	NS
BMI (kg/m^2^)	29.9 ± 4.5	29.3 ± 4.1	NS
Type of ACS n(%):			NS
- Anterior MI	10 (32.2 %)	10 (38 %)	
- Inferior MI	18 (58.0 %)	16 (52 %)	
- Lateral MI	2 (6.5 %)	0 (0 %)	
- Unstable angina	1 (3.3 %)	0 (0 %)	
Heart rate (beats/min)	66 ± 11	63 ± 15	NS
Systolic Blood Pressure (mmHg)	119 ± 18	123 ± 17	NS
Diastolic Blood Pressure (mmHg)	74 ± 12	76 ± 12	NS
Antidiabetic treatment at inclusion n(%):			NS
• metformin	9 (29 %)	11 (42 %)	
• Sulfonylurea or glinide	11 (35 %)	15 (58 %)	NS
• acarbose	1 (3 %)	1 (4 %)	NS
• DPP-4 inhibitor	1 (3 %)	2 (8 %)	NS
• pioglitazone	0 (0 %)	2 (8 %)	NS
• insulin	17 (55 %)	15 (58 %)	NS
Cardiovascular drugs n(%):			
• ACE inhibitor/ARB	28 (90 %)	24 (92 %)	NS
• Beta-blocker	26 (84 %)	22 (85 %)	NS
• Antiplatelet agents	31 (100 %)	26 (100 %)	NS
• statin	29 (94 %)	25 (96 %)	NS
Baseline HbA1c (%)	8.2 ± 1.4	8.4 ± 1.2	NS
Baseline fructosamine (μmol/l)	274 ± 45	278 ± 52	NS
Baseline fasting glucose (mg/dl)	152 ± 42	164 ± 104	NS
Baseline VO2 peak (ml/kg/min)	17.4 ± 4.7	16.5 ± 3.8	NS
Baseline ventilatory threshold (ml/kg/min)	12.6 ± 4.0	12.2 ± 3.9	NS

Data are means ± SD or n (%)

*CHD* coronary heart disease, *ACS,* acute coronary syndrome, *MI* myocardial infarction, *ACE* angiotensin-converting-enzyme, *ARB* angiotensin receptor blocker

### Results of CR in the whole studied population and in each treatment group

For the whole diabetic population studied, the mean gain in VO2 peak after CR was 2.7 ± 2.5 ml/kg/min (16 ± 15 % in relative value).

Among the 57 patients who completed the study, the compliance was excellent in both groups with a mean attendance rate of 19.6/20 sessions in the control treatment group and of 19.8/20 sessions in the intensive treatment group.

The results of CR for each treatment group are shown in Table 2. The final values of the VO2 peak or the ventilatory threshold as well as the gain in VO2 peak or ventilatory threshold were not different between the 2 treatment groups. Similarly, mean final values of fructosamine at the end of CR were not different between the 2 groups. The mean reduction in plasma fructosamine level was not significantly different between the two groups (−37 ± 46 vs.-31 ± 40 μmol/l, *p* = 0,44). As expected, 100 % of the patients in the intensive treatment group were receiving insulin during CR, whereas 55 % of the patients were on insulin in the control treatment group. In the patients on insulin, the mean insulin dose was not significantly different between the 2 treatment groups. Body weight was not significantly modified after CR in both groups.

**Table 2:** MeanVO2 peak, ventilatory threshold and fructosamine values before and after CR for each treatment group

	Control treatment group (n = 31)	Intensive treatment group (n = 26)	p
Baseline VO2 peak (ml/kg/min)	17.4 ± 4.7	16.5 ± 3.8	NS
End-CR VO2 peak (ml/kg/ min)	20.5 ± 5.4	19.2 ± 5.0	NS
Baseline ventilatory threshold (ml/kg/min)	12.6 ± 4.0	12.2 ± 3.9	NS
End-CR ventilatory threshold (ml/kg/ min)	15.0 ± 5.0	14.3 ± 4.6	NS
Gain in VO2 peak (ml/kg/min)	2.7 ± 2.8	2.6 ± 2.2	NS
Gain in ventilatory threshold (ml/kg/min)	2.2 ± 2.4	2.2 ± 2.5	NS
Baseline fructosamine (μmol/l)	274 ± 45	278 ± 52	NS
End-CR fructosamine (μmol/l)	244 ± 41	240 ± 41	NS
Baseline fasting glucose (mg/dl)	152 ± 42	164 ± 104	NS
End-CR fasting glucose (mg/dl)	133 ± 32	126 ± 25	NS
Patients on insulin during CR	17 (55 %)	26 (100 %)	<0.0001
Mean insulin dose in insulin-treated patients (UI/day)	38 ± 25	44 ± 26	NS
Patients on metformin during CR	12 (39 %)	4 (15 %)	0.051

Data are means ± SD or n (%)

*CR* cardiac rehabilitation

Only 5 minor hypoglycemic events were recorded during the study in the intensive treatment group and no serious hypoglycemic event occurred.

### Results of CR according to the glucose control during CR

**Fig. 1:**
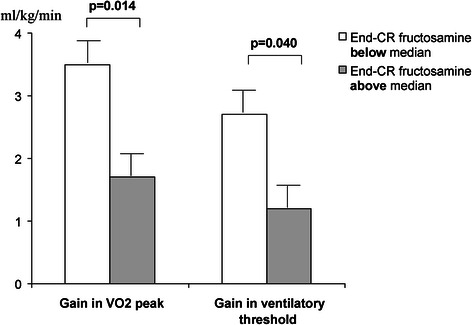
Gain in VO2 peak and ventilatory threshold during CR in patients with final fructosamine level below the median value and in patients with final fructosamine level above the median value

To analyse the effect of glycemic control during CR on the results of CR, we compared the data between two pre-specified groups according to the final fructosamine level. Patients who had a final fructosamine level below the median value (240 μmol/l), indicating good glycemic control during CR, showed, as compared to those with a final fructosamine level above the median value, significantly higher gain in VO2 peak (3.5 ± 2.5 vs. 1.7 ± 2.4 ml/kg/min, *p* = 0.014) and in ventilatory threshold (2.7 ± 2.5 vs. 1.2 ± 1.9 ml/kg/min, *p* = 0.04) as shown in Fig. 1. The percentage of “CR responders” (showing a relative gain in VO2 peak ≥ 16 %) was significantly higher among the patients with final fructosamine below the median value than among those with final fructosamine above the median value (66 % vs. 36 %, *p* = 0.011) (Table 3).

**Table 3:** Characteristics of the patients with final fructosamine below the median value and of the patients with final fructosamine above the median value

	Final fructosamine level below median (n = 29)	Final fructosamine level above median (n = 28)	p
Gender (M/F)	27/2	19/9	0.039
Age (yrs)	59 ± 10	59 ± 8	NS
Diabetes duration (yrs)	7 ± 7	9 ± 8	NS
History of previous CHD	8 (26 %)	7 (26 %)	NS
Tobacco smoking n(%)	10 (34 %)	8 (29 %)	NS
BMI (kg/m^2^)	31.0 ± 4.5	28.6 ± 3.6	0.048
Type of ACS n(%):			NS
- Anterior MI	12 (41.4 %)	8 (28.6 %)	
- Inferior MI	16 (55.2 %)	18 (64.2 %)	
- Lateral MI	1 (3.4 %)	1 (3.6 %)	
- Unstable angina	0 (0 %)	1 (3.6 %)	
Antidiabetic treatment at inclusion n(%):			NS
• Metformin	12 (41 %)	8 (29 %)	
• Sulfonylurea or glinide	14 (48 %)	12 (43 %)	NS
• Acarbose	0 (0 %)	2 (7 %)	NS
• dpp4 inhibitor	1 (3 %)	2 (7 %)	NS
• Pioglitazone	1 (3 %)	1 (4 %)	NS
• Insulin	15 (52 %)	17 (61 %)	NS
Cardiovascular drugs n(%):			NS
• Statin	27 (93 %)	27 (96 %)	
• ACE/inhibitor/ARB	28 (96 %)	24 (86 %)	NS
• Beta-blocker	25 (86 %)	23 (82 %)	NS
• Antiplatelet agents	29 (100 %)	28 (100 %)	NS
Baseline HbA1c (%)	7.9 ± 1.1	8.8 ± 1.4	0.015
Baseline fructosamine (μmol/l)	253 ± 40	297 ± 51	0.0001
Baseline VO2 peak (ml/kg/min)	17.1 ± 4.1	17.1 ± 4.7	NS
Baseline ventilatory threshold (ml/kg/min)	12.4 ± 3.2	12.4 ± 4.8	NS
Final (End-CR) fasting glucose (mg/dl)	119 ± 21	152 ± 32	0.005
Final (End-CR) HbA1c (%)	6.8 ± 0.9	7.9 ± 1.0	<0.0001
Final (End-CR) fructosamine (μmol/l)	213 ± 17	274 ± 29	<0.0001
Gain in VO2 peak (ml/kg/min)	3.5 ± 2.4	1.7 ± 2.4	0.014
Gain in ventilatory threshold (ml/kg/min)	2.7 ± 2.5	1.2 ± 1.9	0.040
CR responders n (%)	19 (66 %)	10 (36 %)	0.011
Patients on insulin during CR	22 (76 %)	25 (89 %)	NS
Mean insulin dose in insulin-treated patients (UI/day)	38 ± 24	46 ± 30	NS

*CHD* coronary heart disease; *ACS* acute coronary syndrome, *MI* myocardial Infarction; *ACE* angiotensin-converting-enzyme; *ARB* angiotensin receptor blocker; *CR* cardiac rehabilitation

The characteristics of the patients with final fructosamine below the median value and those with the final fructosamine level above the median value are shown in Table 3. Age, duration of diabetes, history of previous CHD, tobacco smoking, type of ACS, baseline antidiabetic treatment, cardiovascular drug use, baseline VO2 peak, baseline ventilatory threshold, insulin use during CR and mean insulin dose in the insulin-treated patients were similar in the 2 groups. Baseline and final HbA1c as well as baseline fructosamine values were significantly higher in the patients with final fructosamine level above the median value, compared to those with final fructosamine level below the median value. As expected, mean final fructosamine was higher in the patients with final fructosamine level above the median value. Mean BMI was lower and the percentage of women higher in the group of patients with final fructosamine level above the median value.

### Factors influencing gain in VO2 peak during CR

In univariate analysis, gain in VO2 peak after CR was negatively correlated with basal (r = −0.31, *p* = 0.017) and final fructosamine (r = −0.36, *p* = 0.005) and with basal (r = −0.36, *p* = 0.005) and final HbA1c (r = −0.33, *p* = 0.013). Gain in VO2 peak was not correlated with age, duration of diabetes nor with BMI. The mean gain in VO2 peak was not significantly different between men and women (+2.9 ± 2.4 vs. + 1.7 ± 2.7 ml/kg/min, *p* = 0.20). The mean gain in VO2 peak was not different between patients with anterior MI versus those with other location of MI, between patients with and without history of previous CHD, nor between patients who received insulin treatment during CR versus those who did not.

In multivariable analysis, gain in VO2 peak was significantly negatively associated with final fructosamine (β = −357, *p* = 0.010) but not with age, gender, baseline fructosamine, diabetes duration, BMI, basal VO2 peak, type of SCA, treatment group (insulin intensive/control) or insulin treatment during CR (Table 4). When final fructosamine value was replaced in the statistical model by the qualitative variable “fructosamine above the median value” (yes or no), the latter was significantly negatively associated with final fructosamine (β = −342, *p* = 0.014) whereas the other variables were not (Table 4).

**Table 4:** Multivariable analysis with gain in VO2peak as dependent variable

Variables	β	t	p
A. Model 1 with age, gender, duration of diabetes, BMI, baseline fructosamine, final fructosamine, baseline VO2 peak, type of SCA and treatment group (insulin intensive/control) as independent variables			
*Final fructosamine*	*−0.357*	*−2.680*	*0.010*
Age	−0.086	−0.638	0.527
Gender	−0.090	−0.638	0.527
Diabetes duration	−0.033	−0.240	0.812
BMI	−0.092	−0.651	0.518
Baseline fructosamine	−0.167	−1.032	0.307
Baseline VO2 peak	0.123	0.920	0.362
Type of ACS	−0.106	−0.786	0.436
Treatment group (insulin intensive/control)	−0.038	−0.282	0.779
B. Model 2 (similar to model 1 with the variable “Fructosamine above the median” instead of “Final fructosamine”)			
*Fructosamine above median*	*−0.342*	*−2.549*	*0.014*
Age	−0.077	−0.572	0.570
Gender	−0.099	−0.695	0.490
Diabetes duration	−0.040	−0.295	0.769
BMI	−0.083	−0.588	0.559
Baseline fructosamine	−0.207	−1.406	0.166
Baseline VO2 peak	0.099	0.736	0.465
Type of ACS	−0.184	−1.379	0.174
treatment group (insulin intensive/control)	−0.046	−0.338	0.737

β standardized coefficient

*ACS* acute coronary syndrome

Similar results when the variable “Treatment with insulin” is introduced into the model instead of “Treatment group (insulin intensive/control)”

In mutivariate analysis, CR-response (relative gain in VO2 peak ≥16 %) was significantly negatively associated with final fructosamine (Wald = 4.22, *p* = 0.040) but not with age, gender, baseline fructosamine, diabetes duration, BMI, treatment group (insulin intensive/control) or insulin treatment during CR. When final fructosamine value was replaced in the statistical model by the qualitative variable “fructosamine above the median value” (yes or no), the latter was significantly negatively associated with final fructosamine (Wald = 4.86, *p* = 0.028) whereas the other variables were not.

## Discussion

Here we present the results of the DARE study, which is the first intervention study aiming to determine whether good glycemic control during CR may improve the gain in VO2 peak. We demonstrate that patients with good glycemic control during CR show a significantly higher gain in VO2 peak than those with less good glycemic control. In addition, we demonstrate that final fructosamine level, reflecting glycemic control during CR, is an independent factor influencing gain in VO2 peak.

In the present study, only 7 patients did not complete the cardiac rehabilitation program for personal reasons, representing a drop-out rate of 11 %. This is lower than drop-out rates reported in some studies [33, 34], but similar to the drop-out rate stated by *Wittmer et al*. [35].

In order to assess mean glycemic level during CR, we used fructosamine level at the end of the CR program. Indeed, fructosamine, which is an estimate of mean glycemic level during the 2–3 previous weeks [32] is more appropriate to assess mean blood glucose level during the CR period (which was shorter than 8 weeks) than HbA1c, reflecting mean glycemic level during the 3 previous months. In clinical practice, fructosamine level is used in situations such as pregnancy for which short term assessment of mean glycemic level is needed [32].

Patients who had final fructosamine level below the median value showed a significant larger gain in VO2 peak than those having final fructosamine level above the median. It is interesting to note that patients with final fructosamine below the median had a mean final fructosamine level of 213 μmol/l, indicating good glycemic control during CR. In addition, as shown by the multivariate analysis, final fructosamine level is a strong independent determinant for gain in VO2 peak during CR. Moreover, final fructosamine level is shown to be a significant independent predictor of the response to CR. Together, these data indicate that good glycemic control during CR is an important factor contributing to the optimal gain in VO2 peak independently of other factors including baseline glucose control as demonstrated by the multivariate analysis. We have to note that patients who had final fructosamine level below the median value showed a slightly higher BMI than those who had final fructosamine level above the median value. We have no clear explanations for that. However, this difference in BMI between the 2 groups is not likely to have influenced the gain in VO2 peak since BMI has been taken into account in the multivariate analysis demonstrating the independent association between good glycemic control during CR and gain in VO2 peak.

When comparing the patients included in the intensive treatment group with those included in the control group, we did not find any difference in gain in VO2 peak during CR. This is likely to be due to the fact that glycemic control was identical between the 2 groups. This reinforces the data of our multivariate analysis showing that final fructosamine, assessing glucose control during CR, is a significant independent factor associated with gain in VO2 peak, when insulin treatment is not. This indicates that good glycemic control during CR influences positively the gain in VO2 peak whatever the antidiabetic treatment used.

Although some studies did not report reduced efficacy of CR on exercise capacity in patients with diabetes [36, 33], several other studies have shown that CR is less effective in patients with diabetes [21–24]. These discrepancies could be due to differences in ethnicity, in glycemic control and in control groups. In a previous study, we found that fasting blood glucose was an independent factor influencing gain in VO2 peak during CR [24]. In the present prospective study, we demonstrate that good glycemic control during CR improves significantly the gain in VO2 peak. Our results are in line with a recent study which showed a negative correlation between HbA1c and improvement in knee extensor muscle strength in patients undergoing CR after coronary artery bypass graft surgery [37] and with another one which reported an independent negative association between HbA1c and exercise capacity, during exercise stress testing, in patients with type 2 diabetes [38]. All together, these data give emphasis to the detrimental effect of hyperglycaemia on the improvement of exercise capacity during CR. Accumulating evidence suggests the unfavourable effect of hyperglycemia on cardiomyocytes and muscles that may participate in changes on exercise capacity (VO2). Indeed, hyperglycemia has been shown, *in vitro*, to increase reactive oxygen species in the cardiomyocytes leading to myocardial apoptosis [39–41]. Hyperglycemia also stimulates myocardial endoplasmic reticulum (ER) stress which has been shown to promote cardiomyocyte dysfunction and to contribute to cardiomyopathy in diabetic rats [42, 43]. Several studies have shown impairment of cardiomyocyte calcium cycling induced by hyperglycemia [44–46]. *In vitro*, cardiomyocytes maintained in a high glucose concentration culture medium exhibit slower cytosolic Ca^++^ clearing, prolonged action potentials and prolonged relaxation after only one day [44]. Hyperglycemia has also been shown, *in vitro*, to increase the O-GlcNAcylation of nuclear proteins in the cardiomyocytes leading to impair calcium cycling [46]. Furthermore, in culture myocytes, high glucose promotes the degradation of the transcription factor GATA4 essential for cardiomyocyte growth and survival [47]. Moreover, it has been demonstrated that hyperglycemia reduces the expression of caveolin-3 in the cardiomyocytes through protein kinase C β2 activation leading to diastolic cardiac dysfunction [48]. Interestingly, control of plasma glucose attenuates oxidative stress and slows the progression of heart failure in mice [49].

In addition to cardiomyocyte dysfunction, skeletal muscle dysfunction induced by hyperglycemia may also play a role. Indeed, an impaired functional capacity of mitochondria in skeletal muscle is observed in type 2 diabetes [50]. In addition, decreased skeletal muscle strength has been reported in patients with diabetes, directly correlated with HbA1c [37].

Glycation of haemoglobin, itself, could also play a role in reduced gain in VO2 peak in patients with uncontrolled diabetes. Indeed, it has recently been shown that patients with type 1 diabetes free from clinical micro- and macro-angiopathy but with poor glycemic control showed during exercise lower VO2 peak and a blunted deoxyhaemoglobin (HHb) increase, indicating lower muscle oxygen extraction, suggesting higher oxygen affinity of glycated haemoglobin [51].

We note that in our present study intensive treatment with insulin is not superior to the usual antidiabetic treatment. This indicates that in type 2 diabetes, intensive insulin treatment is not suited for all patients. This is likely due to the fact that several patients with type 2 diabetes show marked insulin resistance with poor response to insulin treatment. This is the reason why it is suggested to adopt, in each patient with type 2 diabetes, a personalized treatment based on the pathophysiological profile of his diabetes [52].

It has been demonstrated that increased VO2 peak is associated with decreased cardiovascular and all-cause mortality [16, 17]. In a long-term prognosis study performed in men with coronary disease, it has been shown that a 1-ml/kg/min increment in VO2 peak leads to a 9 % reduction of mortality [17]. This improvement of VO2 peak is an important target in CR in order to reduce mortality. The results of the DARE study clearly show that good glycemic control during CR is important to optimize the gain in VO2 peak. Thus, it seems important to pay attention to glycemic control of patients with diabetes during CR in order to improve their long-term prognosis.

One limitation of our study is that the results are limited to a Caucasian population. Additional studies in non Caucasian populations are needed to know whether our results may be extrapolated to all ethnic groups.

## Conclusions

The DARE study shows that fructosamine level at the end of the CR-program is an important determinant of gain in VO2 peak in patients with type 2 diabetes and that good glycemic control in CR is associated with significantly better gain in VO2 peak, independent of the treatment used (insulin or not). These data indicate that good glycemic control of type 2 diabetes in CR, after myocardial infarction, is mandatory in order to get optimal gain in VO2 peak.
